# Environmental Drivers of Habitat Suitability for Shortbill Spearfish (*Tetrapturus angustirostris*) in the Pacific Ocean: A Comparison of Single and Ensemble Models

**DOI:** 10.3390/ani16172772

**Published:** 2026-09-03

**Authors:** Yiwei Yang, Jiaqi Wang, Heyang Huang, Yanan Li, Feng Wu, Siquan Tian

**Affiliations:** 1 College of Marine Living Resource Sciences and Management, Shanghai Ocean University, Shanghai 201306, China; 17718180497@163.com (Y.Y.); i_shqyvc@163.com (H.H.); ynli@shou.edu.cn (Y.L.); fwu@shou.edu.cn (F.W.); sqtian@shou.edu.cn (S.T.); 2 National Distant-Water Fisheries Engineering Research Center, Shanghai Ocean University, Shanghai 201306, China; 3 Key Laboratory of Sustainable Exploitation of Oceanic Fisheries Resources, Ministry of Education, Shanghai 201306, China

**Keywords:** shortbill spearfish, habitat suitability, ensemble modelling, environmental preference, Pacific tuna longline fishery

## Abstract

Understanding habitat suitability and environmental preferences of shortbill spearfish (*Tetrapturus angustirostris*) is essential for assessing and mitigating bycatch risk in pelagic longline fisheries. Using observer data collected by the Chinese Pacific tuna longline fishery, including 19,932 longline sets collected during 2010–2021, we compared five individual modelling algorithms and three ensemble strategies to predict habitat suitability across the Pacific Ocean and identify key environmental drivers. Model performance was evaluated using repeated 10-fold cross-validation with three repetitions. The stacking ensemble achieved the highest predictive performance (AUC = 0.872; TSS = 0.591), followed closely by random forest (RF; AUC = 0.869), although the absolute difference between these two models was small. A sensitivity analysis including log-transformed observed hooks resulted in only minor improvements in RF and stacking performance and did not materially alter the main habitat-suitability patterns. Both generally outperformed the other individual and weighted ensemble models. Permutation-based importance analyses consistently identified salinity as the strongest environmental predictor of habitat suitability, followed by chlorophyll-a concentration, distance to shore, and dissolved oxygen concentration. Partial dependence analyses showed that the stacking ensemble produced smoother and more readily interpretable responses to environmental gradients, particularly salinity and dissolved oxygen, whereas RF placed greater importance on a limited number of dominant predictors. Highly suitable habitat was primarily predicted in subtropical offshore waters of the southeastern Pacific (10° S–25° S, 100° W–130° W), which were characterized by relatively high salinity, moderate dissolved oxygen concentrations, and low chlorophyll-a concentrations. A secondary area of elevated suitability was identified in the central North Pacific (160° E–180° E, 15° N–25° N). Overall, the stacking ensemble provided robust predictions of shortbill spearfish habitat suitability, whereas RF remained useful for identifying dominant environmental correlates. The predicted habitat maps provide a spatial basis for identifying potential bycatch-risk hotspots and supporting adaptive management strategies, such as spatially targeted monitoring and mitigation measures in tuna longline fisheries.

## Simple Summary

The shortbill spearfish is a large oceanic fish that can be accidentally caught in tuna longline fisheries. Because this species has received less scientific attention than many target fishes, limited information is available on where it is most likely to occur and which ocean conditions are associated with its distribution. This study used fishery observer records from the Chinese Pacific tuna longline fishery to predict areas where shortbill spearfish habitat is more suitable. We compared several habitat models and found that the stacking ensemble model performed best overall, while random forest also performed well. We also found that the two models showed some differences in how they responded to ocean conditions such as sea surface salinity, dissolved oxygen, distance from shore, and chlorophyll-a concentration. Highly suitable areas were mainly located in offshore subtropical waters of the southeastern Pacific, with another suitable area in the north-central Pacific. Sea surface salinity was the strongest environmental indicator. These habitat maps can help identify areas where accidental catches may be more likely, supporting better monitoring and management of tuna longline fisheries.

## 1. Introduction

Billfishes are ecologically important apex predators in pelagic ecosystems, and they represent both target catches and incidental catches in longline fisheries. This group comprises target species such as swordfish (*Xiphias gladius*) and blue marlin (*Makaira nigricans*), as well as commonly captured bycatch species including shortbill spearfish (*Tetrapturus angustirostris*) and striped marlin (*Kajikia audax*). These species are of considerable ecological and management interest. Compared with target species, bycatch billfishes often receive less systematic monitoring and management assessment, despite the fact that they are continuously exposed to fishing mortality from longline and other fisheries [1]. No clear evidence of stock depletion has been reported for shortbill spearfish; its population dynamics, habitat distribution, and responses to fishing pressure remain poorly understood [2]. This discrepancy between actual potential fishing exposure and the current scarcity of relevant ecological research indicates that the status of shortbill spearfish warrants more targeted attention within the framework of global bycatch governance. Investigations into the habitat distribution of shortbill spearfish can effectively fill the aforementioned knowledge gaps, and further provide a scientific foundation for identifying regions with high bycatch risk and formulating precautionary management strategies.

Fish distribution is closely associated with habitat suitability, and temporal and spatial variation in environmental conditions can influence habitat selection and migration pathways [3]. Sea surface temperature, salinity, and chlorophyll-a concentration may affect individual physiology and prey distributions, thereby shaping billfish habitat patterns. The shortbill spearfish is a highly migratory epipelagic species that is widely distributed in tropical and subtropical waters of the Indian and Pacific Oceans. It occupies a broad depth range (0–1830 m) but typically occurs above the thermocline [1]. Previous studies have identified sea surface temperature, mixed-layer depth, and dissolved oxygen as important environmental drivers of billfish habitat distribution [4]. Temperature is considered a major determinant of geographic range, whereas dissolved oxygen and mixed-layer depth may influence vertical habitat use and seasonal movements. Sea surface height and salinity can also contribute to large-scale spatial patterns of juvenile blue marlin and white marlin [5]. However, dedicated studies on the habitat suitability of the shortbill spearfish remain limited, and its specific environmental preferences and primary driving factors in Pacific tuna longline fishing grounds have not been systematically quantified to date.

Species distribution models (SDMs) have been widely used to relate species occurrences to environmental variables and to predict spatial distributions [6]. In recent years, machine-learning algorithms, including random forest, neural networks, and XGBoost, have been increasingly applied to predict the distributions of oceanic fishes [7]. Thoya et al. used boosted regression trees to examine habitat–environment relationships for six billfish species in the Indian Ocean, and identified temperature, mixed-layer depth, and dissolved oxygen as the primary predictors [4]. Rooker et al. used generalized additive models to investigate associations between the distributions of juvenile blue marlin and white marlin (*Kajikia albida*) and environmental conditions [5]. Most previous studies have relied on individual models, whereas systematic comparisons with ensemble methods remain limited. Although machine-learning approaches can improve predictive performance relative to conventional regression models, algorithms differ in their sensitivity to outliers, extrapolation behaviour, generalization capacity, and susceptibility to overfitting [8]. Ensemble methods integrate complementary base learners and may improve generalization in complex modelling tasks [9], and this advantage has been supported by recent large-scale SDM comparisons [10,11]. However, their advantages are not universal: ensemble models can underperform well-tuned individual algorithms when errors accumulate across component models [12]. Marmion et al. found that, although ensemble approaches had higher average predictive accuracy, individual models could match or exceed their performance for particular datasets [13]. Thus, the relative performance of individual and ensemble models should be evaluated empirically for each study system.

Studies on the habitat distribution of billfish commonly rely on several types of species-distribution data. Fishery-dependent data, including catch records, catch per unit effort (CPUE), and tag-recapture information from longline and recreational fisheries, are frequently used to describe broad-scale occurrence patterns and fishery-associated distribution characteristics [14]. Satellite tagging data, particularly those obtained from pop-up satellite archival tags, provide more direct information on horizontal movements, vertical habitat use, and migration routes of billfish, and are therefore valuable for interpreting habitat selection and movement ecology [15]. These biological and fishery data are usually combined with oceanographic variables derived from remote sensing or reanalysis products, such as sea surface temperature, mixed-layer depth, chlorophyll-a concentration, dissolved oxygen, salinity, sea surface height, and other indicators of water-mass structure or productivity, to identify environmental conditions associated with habitat suitability [16]. Among these data sources, bycatch records from tuna longline observer programs are especially useful for documenting interactions between billfish and fishing operations. However, such records are typically characterized by a high proportion of zero-catch observations, uneven spatial and temporal coverage, and dependence on the distribution of fishing effort. These characteristics may constrain model performance and require cautious interpretation of species–environment relationships and predicted habitat suitability.

Although habitat modelling has been increasingly applied to billfish and other pelagic fishes, the available evidence is uneven among species. Previous studies have examined the habitat distribution, environmental associations, and vertical movements of commercially important or better-studied billfish species, including swordfish, blue marlin, striped marlin, and sailfish. These studies have identified variables such as sea surface temperature, mixed-layer depth, dissolved oxygen, chlorophyll-a concentration, and mesoscale oceanographic features as important predictors of billfish distribution [4,5,15]. In contrast, species-specific habitat modelling studies on shortbill spearfish remain limited. Its environmental preferences, potential suitable habitats, and bycatch-risk areas in Pacific tuna longline fishing grounds have not been systematically quantified. Therefore, distinguishing shortbill spearfish from the broader billfish literature is necessary for evaluating whether environmental associations identified for other billfish species can be generalized to this less-studied species.

Accordingly, we used observer data from the Chinese Pacific tuna longline fishery to compare the performance of multiple individual and ensemble models in characterizing environmental associations and predicting habitat suitability for shortbill spearfish. Specifically, we aimed to identify key environmental drivers, delineate potential suitable habitat, and evaluate the implications of model choice for bycatch-risk assessment and conservation management.

## 2. Materials and Methods

### 2.1. Fishery Data

Data on shortbill spearfish bycatch were obtained from the Chinese Pacific tuna longline fishery observer programme during 2010–2021. Scientific observers were trained to collect fishery-dependent information relevant to tuna and other pelagic fish populations. Observers were assigned to longline vessels operating in fishing grounds and recorded fishing dates, locations, operation times, fishing effort, and catches of target and non-target species, together with relevant biological information. They also documented catches of target and non-target species, capture condition, and biological measurements [17]. Because complete observation of every hook within a set was generally not feasible, observers randomly selected a proportion of baskets from each set for catch observation and biological measurements. Therefore, the number of hooks observed within each set reflected the observer sampling effort based on randomly selected baskets.

**Figure 1:**
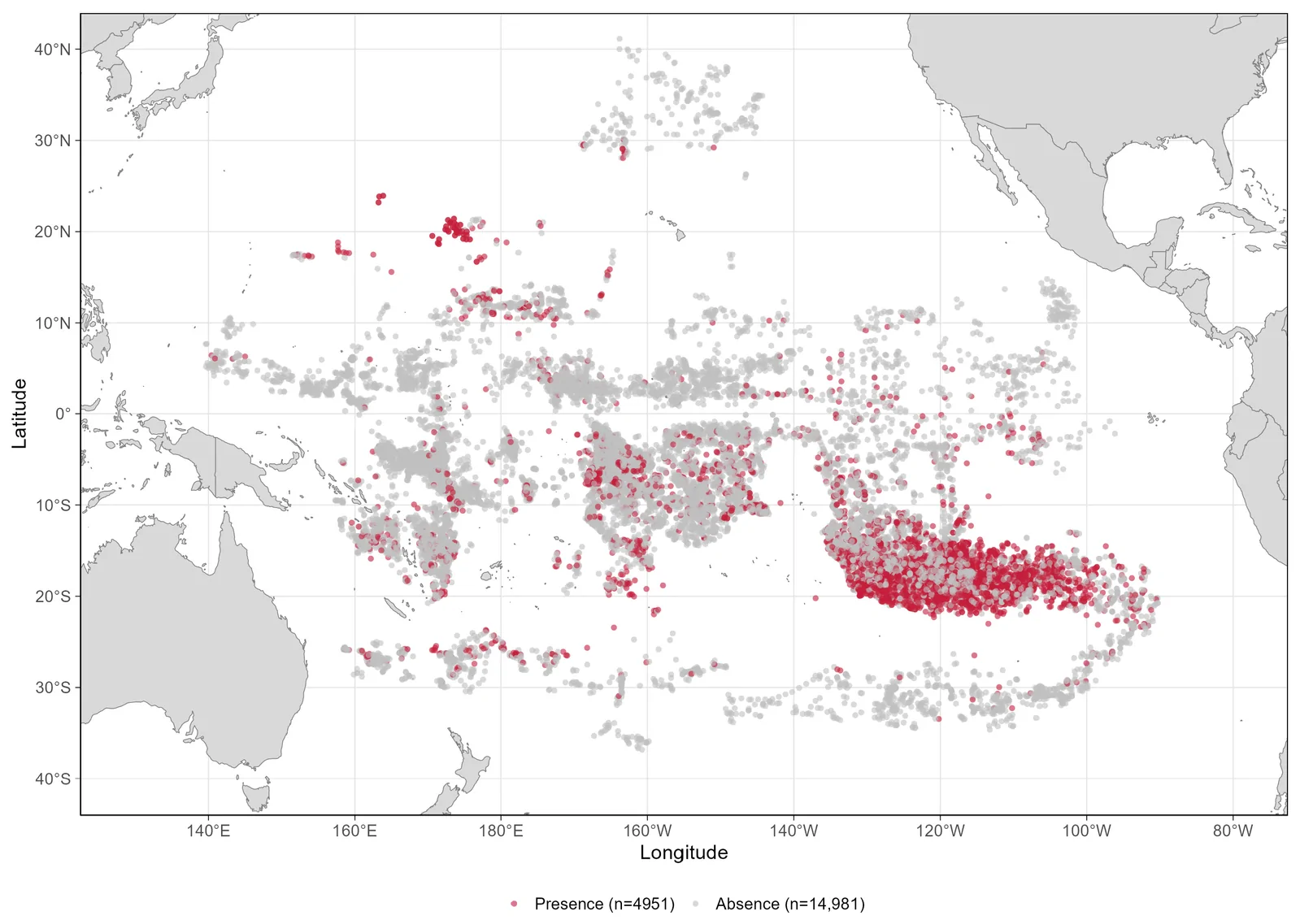
Spatial distribution of longline sets with presence or absence of *Tetrapturus angustirostris* occurrence in the Pacific Ocean.

For the observer dataset, records with missing geographic coordinates, invalid fishing dates, or incomplete occurrence information were removed before model construction. After this quality-control procedure, the final dataset contained 19,932 longline sets collected across 190 observer units, each defined by a unique combination of observer, vessel, and year. The number of hooks deployed per set ranged from 1500 to 6000, with a median of 2722 (interquartile range: 2241–3240). The dataset included 4951 longline sets with shortbill spearfish occurrence records and 14,981 sets without captures. The response variable was converted into a binary occurrence format, with presence defined as longline sets where at least one shortbill spearfish individual was captured and absence defined as sets without observed captures. Observed hooks were not included in the primary occurrence models but were subsequently evaluated as an additional predictor in a sensitivity analysis. Therefore, the binary response represents the observed probability of encountering shortbill spearfish during a fishing set under the recorded fishing operations, rather than a fishery-independent estimate of species presence. Sampling locations were distributed across tropical and subtropical regions of the Pacific Ocean, covering major fishing grounds from longitudes 139.6° E to 90.3° W and latitudes 36.8° S to 41.1° N. The spatial distribution of sampling effort is shown in Figure 1.

### 2.2. Environmental Data

Environmental variables were selected on the basis of previous studies of the spatial ecology of oceanic fishes, particularly istiophorids, and factors affecting longline catch rates [18]. The selected variables represent key oceanographic characteristics that may influence the distribution of epipelagic species. Details of the predictor variables are provided in Table 1. The surface environmental data corresponding to each longline set were obtained from the Copernicus Marine Environment Monitoring Service (CMEMS; https://marine.copernicus.eu/). Bathymetric depth and distance to the nearest land were obtained from the environmental data layers provided by Global Fishing Watch (GFW; https://globalfishingwatch.org/) and extracted for each observed longline set location. For each observed set, environmental variables were matched to the corresponding fishing date and month, and values from the same month and year as the observation were extracted. Environmental values were extracted from the corresponding raster cell using the nearest-neighbour approach.

### 2.3. Statistical Analysis

#### 2.3.1. Environmental Variable Screening

Explanatory variables were screened for multicollinearity using Pearson correlation coefficients and variance inflation factors (VIFs) [19]. An absolute Pearson correlation coefficient of ∣r∣ ≥ 0.7 was used as the threshold for identifying strongly correlated predictor pairs, as this value has been suggested as a practical indicator of collinearity that may distort model estimation and prediction [20]. Therefore, when two variables showed correlations exceeding this threshold, one variable was removed to reduce redundancy among explanatory variables and improve model interpretability. We then retained variables with VIF values < 5 [21]. Variables that satisfied both criteria were used in subsequent model analyses.

#### 2.3.2. Model Selection and Ensemble Construction

Species distribution models were developed in R using biomod2 as the principal modelling framework. Five representative classification algorithms were selected as individual models: random forest (RF), extreme gradient boosting (XGBoost), generalized linear model (GLM), generalized additive model (GAM), and k-nearest neighbours (KNN) [17,22]. These algorithms represent machine-learning, parametric regression, and non-parametric approaches. Model parameters were predefined before model training based on commonly used settings in species distribution modelling and previous applications of these algorithms. The selected parameters were kept consistent across models to facilitate comparison of algorithmic performance. Before model fitting, continuous environmental predictors were standardized using the mean and standard deviation calculated from the training data. The same transformation parameters were subsequently applied to validation data during cross-validation.

Random forest constructs multiple decision trees and aggregates their predictions. It is well suited to high-dimensional data and can capture complex non-linear relationships while reducing the risk of overfitting [23]. We implemented RF using the rf method in the caret package, with mtry fixed at 1 so that one predictor was randomly selected at each split. All remaining parameters were retained at their default settings.

XGBoost is an efficient implementation of gradient-boosted decision trees that sequentially adds weak learners while optimizing a regularized objective function [24]. Hyperparameters were set to nrounds = 200, max_depth = 6, and eta = 0.1; additional parameters were specified to limit overfitting.

A generalized linear model (GLM) with a binomial error distribution and logit link was fitted as the representative parametric approach. This model is equivalent to logistic regression and estimates parameters by maximum likelihood [25]. Unlike tree-based machine-learning models, a standard GLM does not require tuning of parameters such as tree depth or learning rate; therefore, no tuning grid was applied.

Generalized additive models (GAMs) allow non-linear relationships between predictors and the response to be represented by smooth functions while retaining an additive model structure [26]. We implemented GAMs using penalized splines in the mgcv package (version1.9-1), with GCV.Cp as the smoothing-parameter selection criterion and select = TRUE to enable term selection.

KNN is an instance-based learning method that assigns the class of a new observation according to the classes of its k nearest neighbours in the training set [27]. We set k = 5. All predictor variables were centred and scaled before fitting to prevent differences in measurement scale from influencing the distance calculation.

To integrate information from the individual models, we constructed two weighted ensembles and one stacking ensemble. For weighted ensembles, cross-validation AUC and TSS values were used as weights to calculate weighted averages of the predicted probabilities from the five individual models, yielding AUC-weighted and TSS-weighted ensembles [11]. To reduce the influence of poorly performing base learners on ensemble predictions, only individual models that achieved acceptable discriminatory performance were included in ensemble construction. Specifically, models were retained when their cross-validated AUC exceeded 0.7, and TSS exceeded 0.5. These thresholds were selected because AUC values above 0.7 are commonly interpreted as indicating acceptable discrimination in presence–absence modelling, whereas TSS values above 0.5 indicate useful predictive performance beyond random expectation. This filtering step was applied before constructing the AUC-weighted, TSS-weighted, and stacking ensembles. Stacking then used the predicted probabilities from the retained base models as inputs to a logistic-regression meta-model [28].

#### 2.3.3. Model Performance Evaluation and Analysis

Model discrimination and overall predictive performance were evaluated using the area under the receiver operating characteristic curve (AUC) [29] and the true skill statistic (TSS) [30]. AUC quantifies the ability of a model to distinguish presences from absences, with values closer to 1 indicating stronger discrimination. TSS ranges from −1 to 1 and is less sensitive than overall accuracy to prevalence or sample imbalance, with values closer to 1 indicating better performance. To assess model robustness, we used repeated 10-fold cross-validation with three repetitions [31], thereby reducing sensitivity to a particular random partition of the data. To ensure reproducibility, a fixed random seed was set before model training and cross-validation. The same seed was used for data partitioning, model fitting, and ensemble construction.

Overall differences in AUC among models were assessed using the Friedman test. When the overall test was significant, pairwise comparisons were conducted using Wilcoxon signed-rank tests with Bonferroni adjustment for multiple comparisons [32].

#### 2.3.4. Habitat Suitability Analysis

Variable importance was assessed for both RF and stacking models using a common model-agnostic permutation procedure [23,33]. For each held-out validation set, predictor importance was quantified as the decrease in AUC after independently permuting that predictor while retaining all other variables unchanged. For RF, predictions were generated directly after permutation. For stacking, the permuted environmental data were propagated through all fitted base learners, and their updated predictions were subsequently combined by the logistic-regression meta-model [28]. Thus, stacking importance reflected the contribution of each original environmental predictor to the final ensemble prediction rather than the contribution of individual base models [34]. For graphical presentation of within-model ranking patterns only, importance values were additionally rescaled to a 0–100 range separately for each model.

Partial dependence plots (PDPs) were used to examine the marginal association between each environmental predictor and the predicted probability of occurrence. For each predictor, PDPs vary the focal variable across its observed range while retaining the original distribution of the remaining variables, and then calculate the average model response. The resulting curves show the relationship between the predicted occurrence probability of shortbill spearfish and each environmental variable. Because the stacking ensemble did not provide a direct analytical prediction function, we approximated its PDPs using a local-averaging approach based on neighbouring samples [35].

Prediction uncertainty was quantified using model-specific approaches. For the RF model, prediction ambiguity was characterized using the Bernoulli variance, *p* × (1 − *p*), which is highest for probabilities near 0.5 and lowest near 0 or 1. For the stacking ensemble, model disagreement was derived as the variance among predicted probabilities from the fitted base learners before meta-model weighting, thereby capturing inter-model variability within the ensemble [36]. The spatial consistency of habitat-suitability predictions among models was quantified by using Schoener’s D index [37], suitable-habitat area, centroid displacement, spatial patterns of prediction differences, and correlations between predicted values. These metrics were used to compare models in terms of spatial overlap, habitat extent, displacement of distribution centres, and geographic structure of prediction differences. Predicted occurrence probability was classified as unsuitable (<0.2), low suitability (0.2–0.4), moderate suitability (0.4–0.6), or high suitability (≥0.6) [38]. For map visualization, continuous predicted occurrence probabilities were displayed using 0.1 probability intervals to show finer spatial gradients in habitat suitability. The four-class suitability system was used only for class-based summary analyses, including suitable-area estimation, high-suitability habitat overlap, and centroid calculations. Centroids of high-suitability habitat were calculated from high-suitability grid cells weighted by their predicted probabilities, and Euclidean distances between centroids were used to compare the positions of core habitats between models [38]. Consistency between continuous predictions was evaluated using Pearson and Spearman correlation coefficients [39]. The study area was divided into 1° × 1° regular grids, and the mean absolute error (MAE) between model predictions was calculated within each grid and mapped to characterize the spatial heterogeneity of prediction differences.

#### 2.3.5. Sensitivity Analysis of Observer Sampling Effort

To evaluate whether variation in observer sampling effort materially affected model performance and habitat-suitability predictions, an additional sensitivity analysis was conducted for the RF and stacking models. The number of observed hooks per set was log-transformed and included as an additional predictor, while all other predictors and model settings were retained. Models with and without log-transformed observed hooks were compared using AUC and TSS. Consistency between their predictions was further evaluated using Pearson and Spearman correlation coefficients, Schoener’s D, and the Jaccard index for high-suitability habitat (predicted probability ≥ 0.6).

All analyses were conducted in R version 4.4.3 under the Windows operating system. The principal packages used were biomod2 (version4.2-6-2), caret (version7.0-1), raster (version3.6-32), dplyr (version1.1.4), and ggplot2 (version4.0.3) [40].

## 3. Results

### 3.1. Explanatory Variable Screening

**Figure 2:**
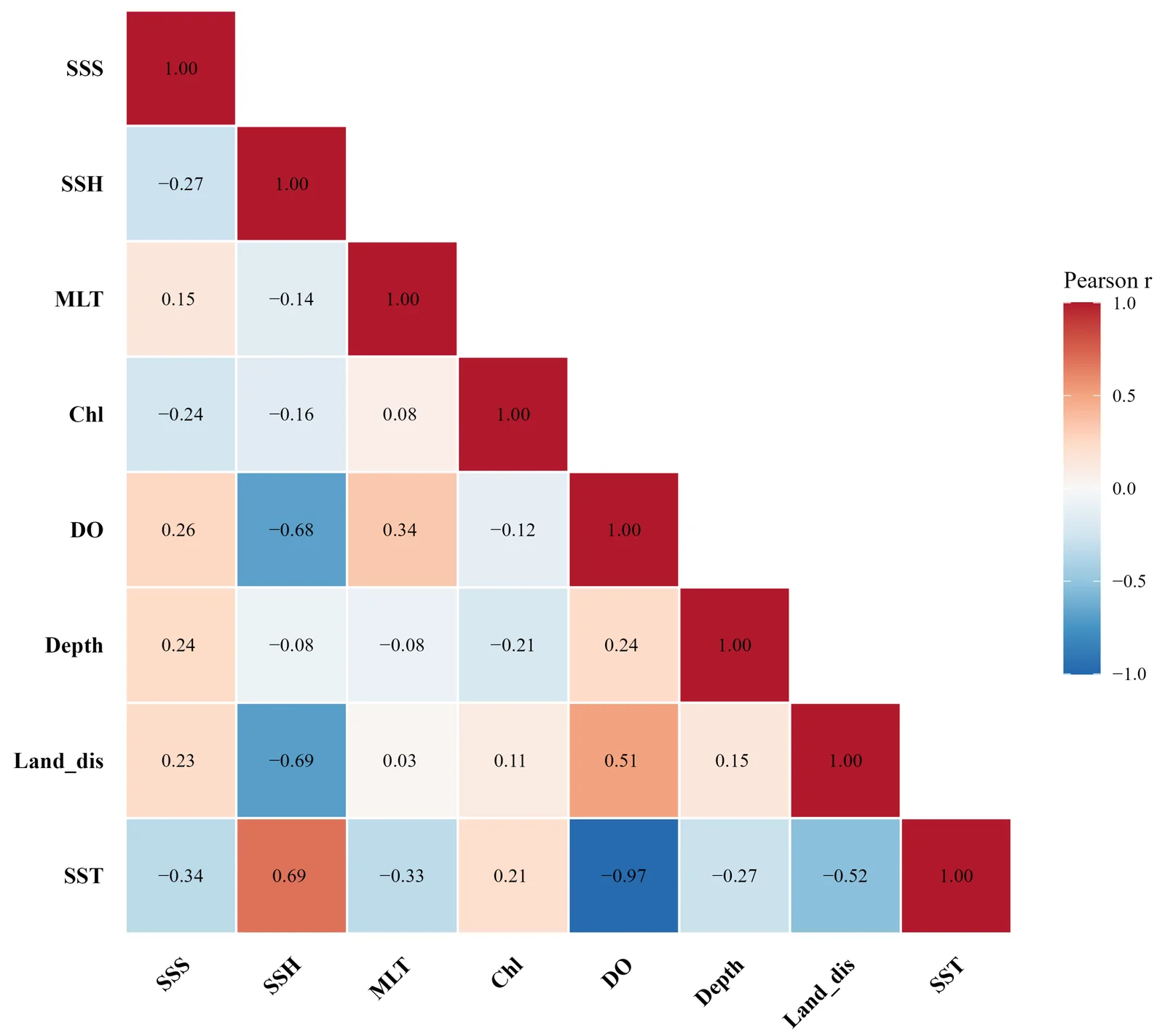
Pearson correlation matrix for environmental predictor variables.

Pearson correlation analysis showed that all pairwise correlations among the explanatory variables were <0.7, except for the correlation between SST and DO, which exceeded 0.7 (Figure 2). To avoid redundant environmental information in the models, DO was retained and SST was excluded from subsequent analyses because DO provides direct information on aerobic habitat availability for pelagic predators, whereas SST partly overlaps with broader hydrographic gradients represented by other retained variables. This decision was made to reduce collinearity while retaining a predictor with clear ecological relevance to habitat suitability.

The initial VIF analysis showed high VIF values for both SST and DO. This pattern was mainly caused by the strong correlation between these two variables. After SST was removed, the VIF values of all retained predictors decreased below 5.0, including DO, whose adjusted VIF declined from 20.10 to 2.67 (Table 2).

### 3.2. Model Performance

**Figure 3:**
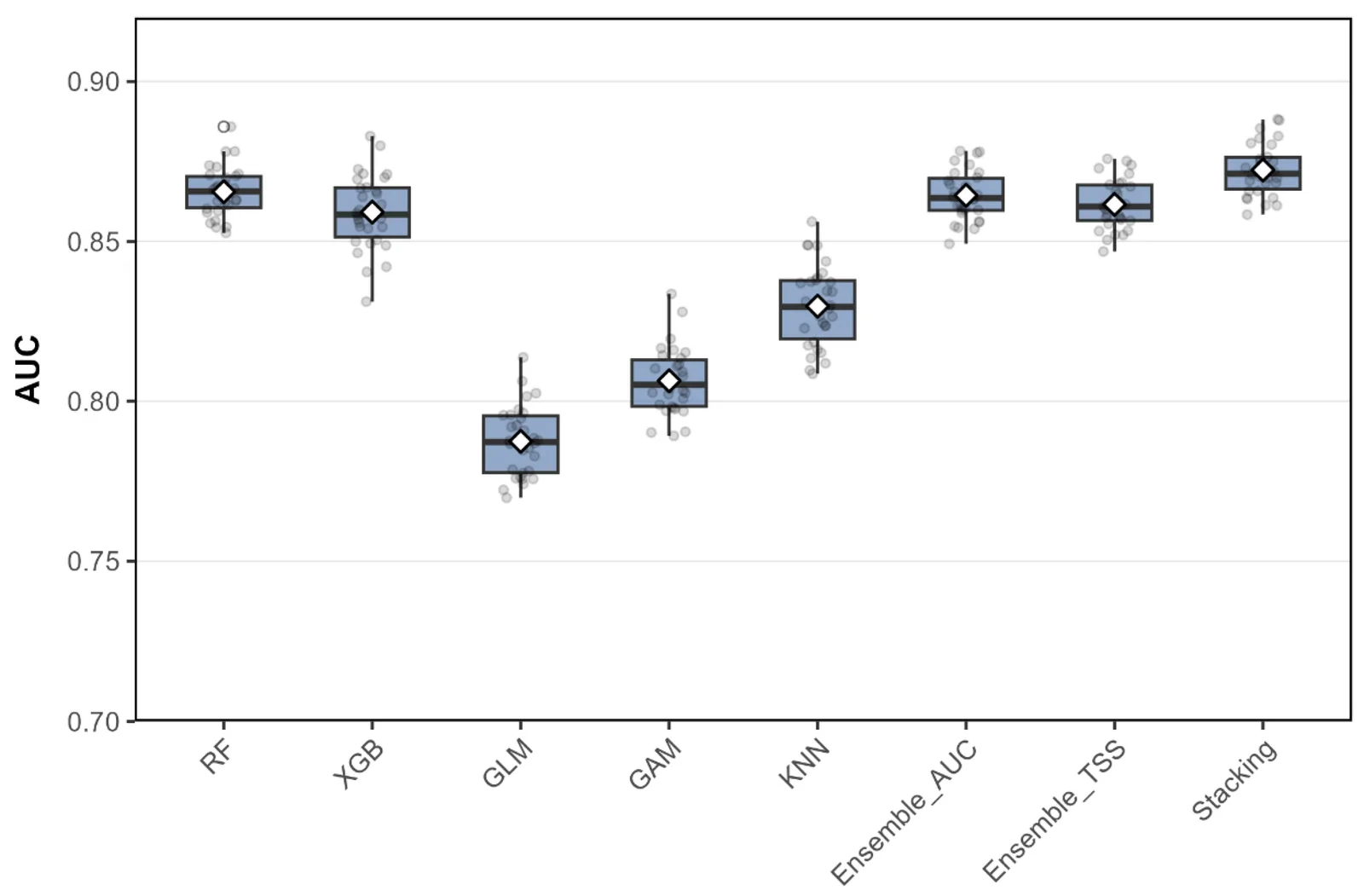
Distribution of cross-validated AUC values for the habitat-suitability models. Note: Boxplots show the median (horizontal line), interquartile range (IQR; box), and 1.5 × IQR (vertical whiskers). Outliers beyond the whiskers are shown as open circles. White diamonds indicate mean values. Small grey points show raw values from each cross-validation replicate. Box width is fixed for visual clarity and does not encode additional statistical information.

**Figure 4:**
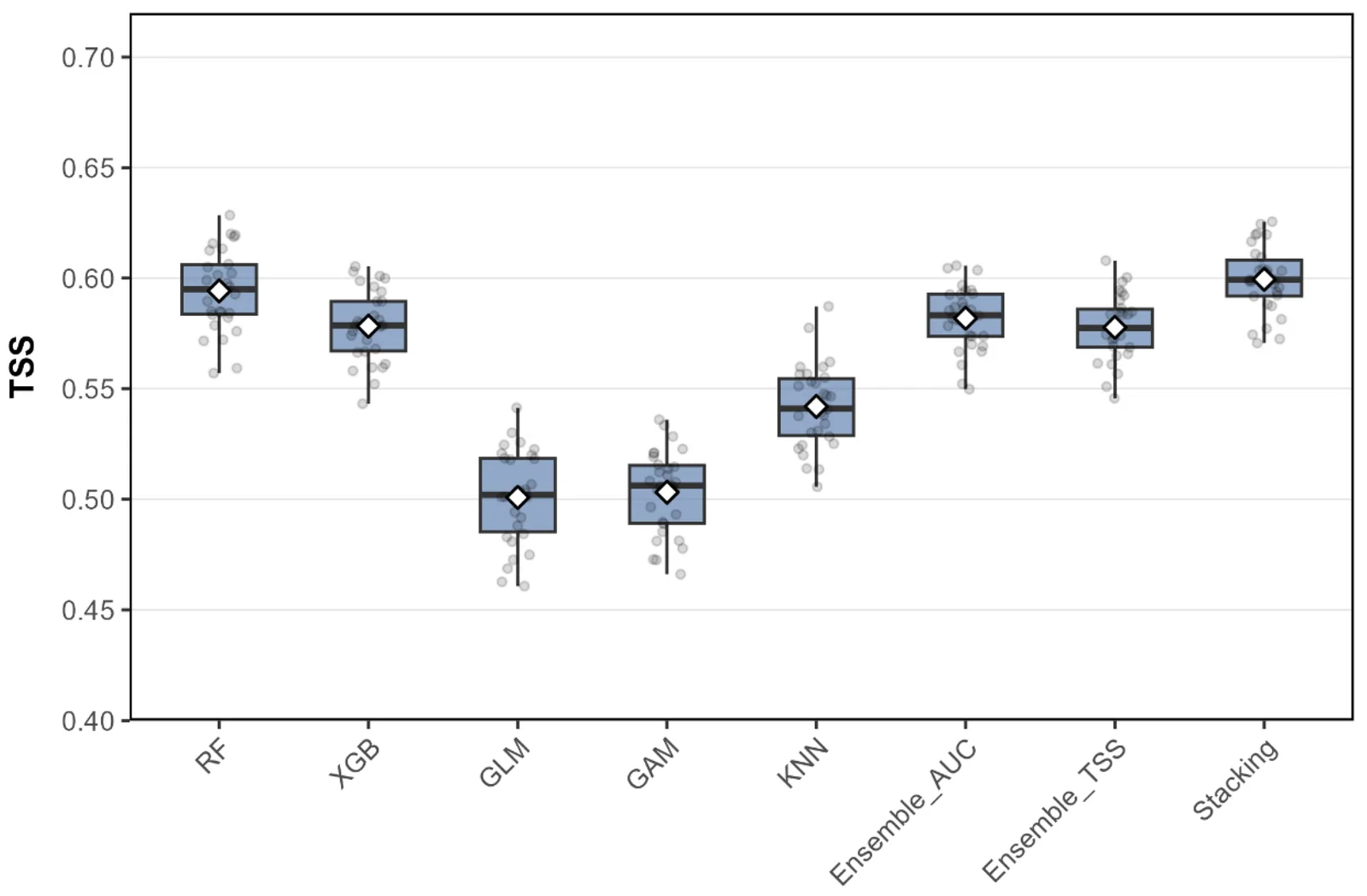
Distribution of cross-validated TSS values for the habitat-suitability models. Note: Boxplots show the median (horizontal line), interquartile range (IQR; box), and 1.5 × IQR (vertical whiskers). Outliers beyond the whiskers are shown as open circles. White diamonds indicate mean values. Small grey points show raw values from each cross-validation replicate. Box width is fixed for visual clarity and does not encode additional statistical information.

The cross-validation revealed differences in predictive performance among the habitat-suitability models. Based on AUC, the stacking ensemble performed best (0.872), followed by RF (0.869), the AUC-weighted ensemble (0.864), and the TSS-weighted ensemble and XGBoost (both 0.861) (Figure 3). The TSS ranking was broadly similar, with stacking again achieving the highest value (0.591) and RF ranking second (Figure 4). Overall, stacking showed the best combined performance, whereas RF was the best-performing individual model and approached the performance of the ensemble models. The two weighted ensemble strategies performed similarly and generally outperformed most individual models. The Friedman test indicated a significant overall difference in AUC among models (χ^2^ = 213.0, *p* < 0.001). Pairwise Wilcoxon tests showed that stacking had significantly higher AUC estimates than the other models (*p* < 0.001), whereas the difference between RF and the AUC-weighted ensemble was not significant (*p* > 0.05).

Although stacking was statistically superior to RF, the absolute difference in AUC was small (0.003), suggesting that the ecological distinction between these two models was limited. Therefore, both models were retained for further comparison of environmental responses and spatial habitat predictions.

### 3.3. Variable Importance

**Figure 5:**
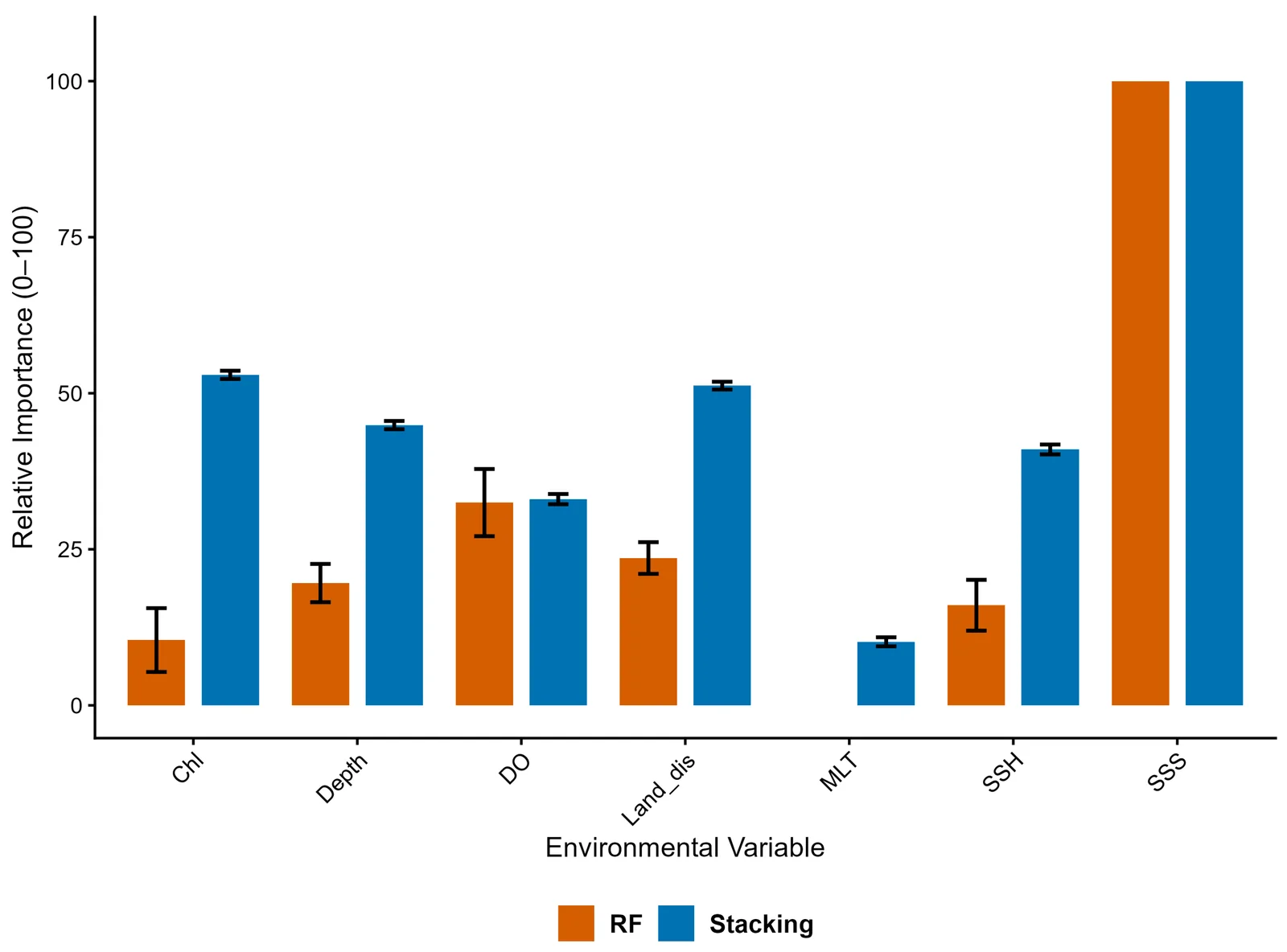
Mean relative permutation importance (0–100) of environmental predictors in the RF and stacking models across repeated cross-validation runs. Error bars represent ± SD. Note: Importance values were calculated using permutation-based importance and then rescaled to a 0–100 range within each model, with 100 representing the most important predictor in that model. Values are shown as mean ± SD across repeated cross-validation runs. For both models, SSS consistently had the highest relative importance (100) in all runs; therefore, its SD was 0, and the error bar is not visible.

Variable-importance analyses identified SSS as the dominant environmental predictor in both RF and the stacking ensemble (Figure 5). Although the ranking of the remaining variables differed between the models, the broad pattern was consistent. In the stacking ensemble, Chl and Land_dis ranked next after SSS, with relative importance scores of 52.86 and 51.70, respectively. In RF, these variables showed moderate importance scores of approximately 26–35. DO remained important in both models and had a slightly higher score in RF (47.74). Depth had a relative importance score of 45.71 in the stacking model, markedly higher than in RF. SSH also had a higher contribution in the stacking model (41.46) than in RF, whereas MLT had no contribution in RF but retained a modest contribution in the stacking model (11.77). Overall, the stacking ensemble distributed importance more evenly across predictors, whereas RF concentrated importance on a smaller number of factors, particularly SSS and DO.

### 3.4. Environmental Response

**Figure 6:**
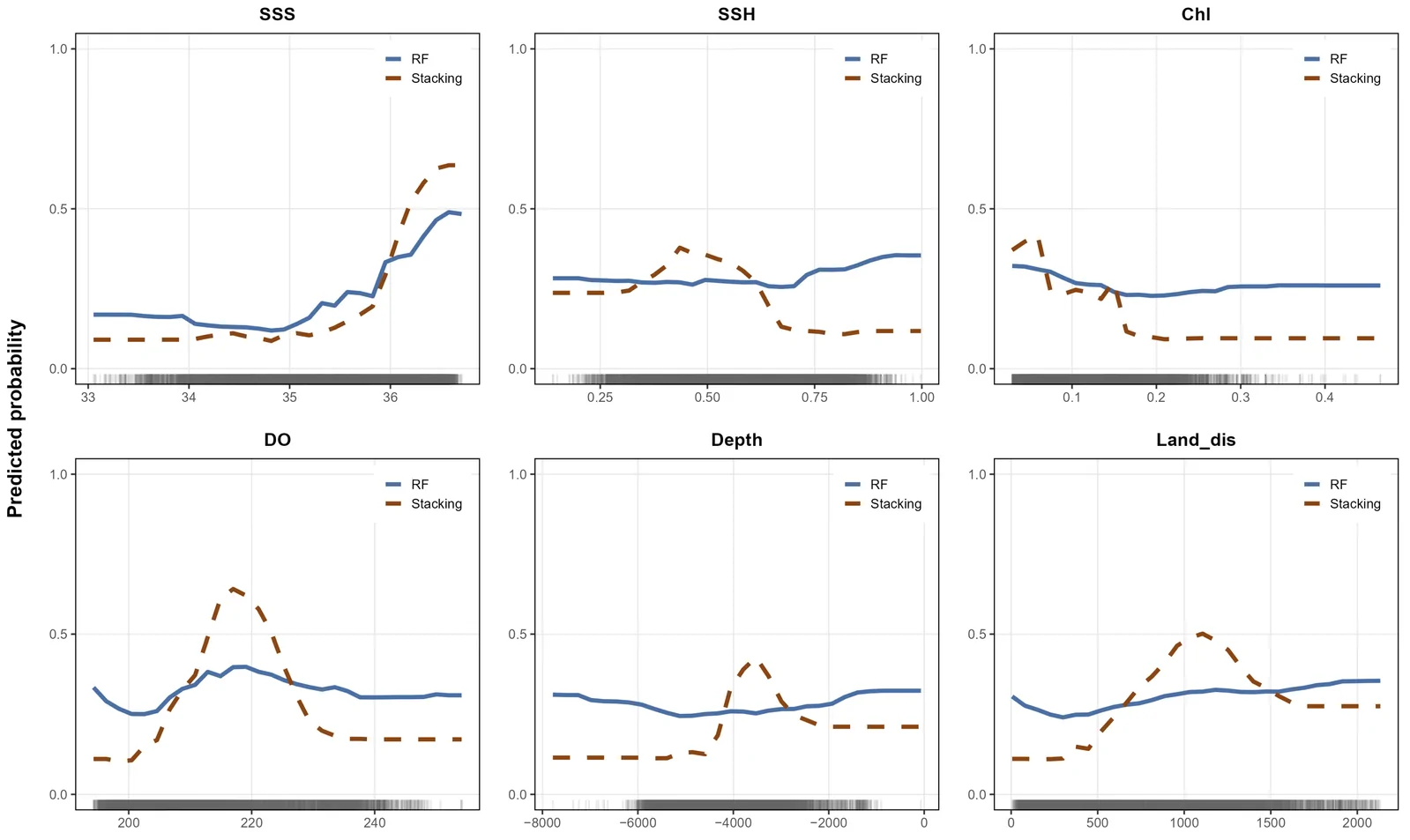
Partial dependence curves for key environmental predictors in the RF and stacking ensemble models.

Partial dependence curves showed broadly consistent response directions between RF and the stacking ensemble, although peak positions, gradient steepness, and the magnitude of local variation differed (Figure 6).

For SSS and Chl, the two models showed similar overall response directions, whereas the stacking ensemble produced more coherent curves with clearer threshold-like transitions. Both models responded positively to SSS and reached their highest suitability at approximately 35–36 psu, with a higher predicted peak for stacking. Predicted suitability declined with increasing Chl in both models, but the decrease was more pronounced in the stacking ensemble above approximately 0.15 mg/m^3^.

For DO, Land_dis, Depth, and SSH, response shapes differed more markedly between the models. The stacking ensemble generally showed clearer local peaks. Both models exhibited a unimodal response to DO, with suitability peaking at approximately 200–230 mmol/m^3^ before declining; the stacking ensemble showed a higher peak and a steeper response curve. For depth, RF showed an initial decline followed by an increase, with the lowest predicted suitability at approximately 5000 m, whereas the stacking ensemble showed a unimodal response peaking at approximately 3500 m. For distance to shore, RF showed an overall increasing trend with a slight decline near 1500 km, whereas the stacking ensemble peaked near 1000 km and then declined. For SSH, RF changed little across most of the gradient and increased only slightly above 0.75 m, whereas the stacking ensemble showed a distinct peak near 0.5 m followed by a rapid decline. Overall, suitable habitat for shortbill spearfish was associated with high SSS, moderate DO, low Chl, and particular combinations of Depth, Land_dis, SSH, and MLT.

### 3.5. Habitat Suitability

**Figure 7:**
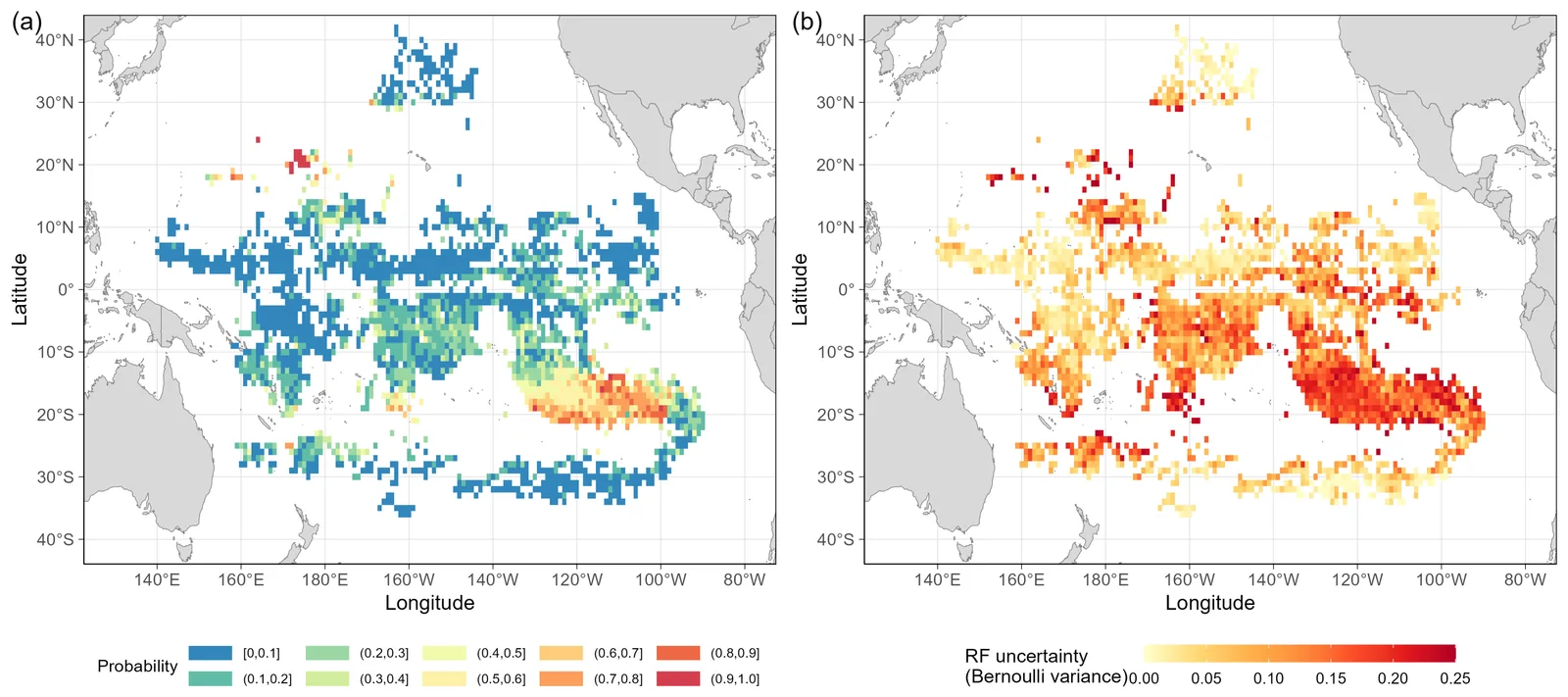
(**a**) Mean predicted habitat suitability and (**b**) prediction ambiguity for *Tetrapturus angustirostris* within 1° × 1° grid cells containing observed longline sets, based on the random forest (RF) model. Predictions were generated only for observed fishing locations and averaged within grid cells.

**Figure 8:**
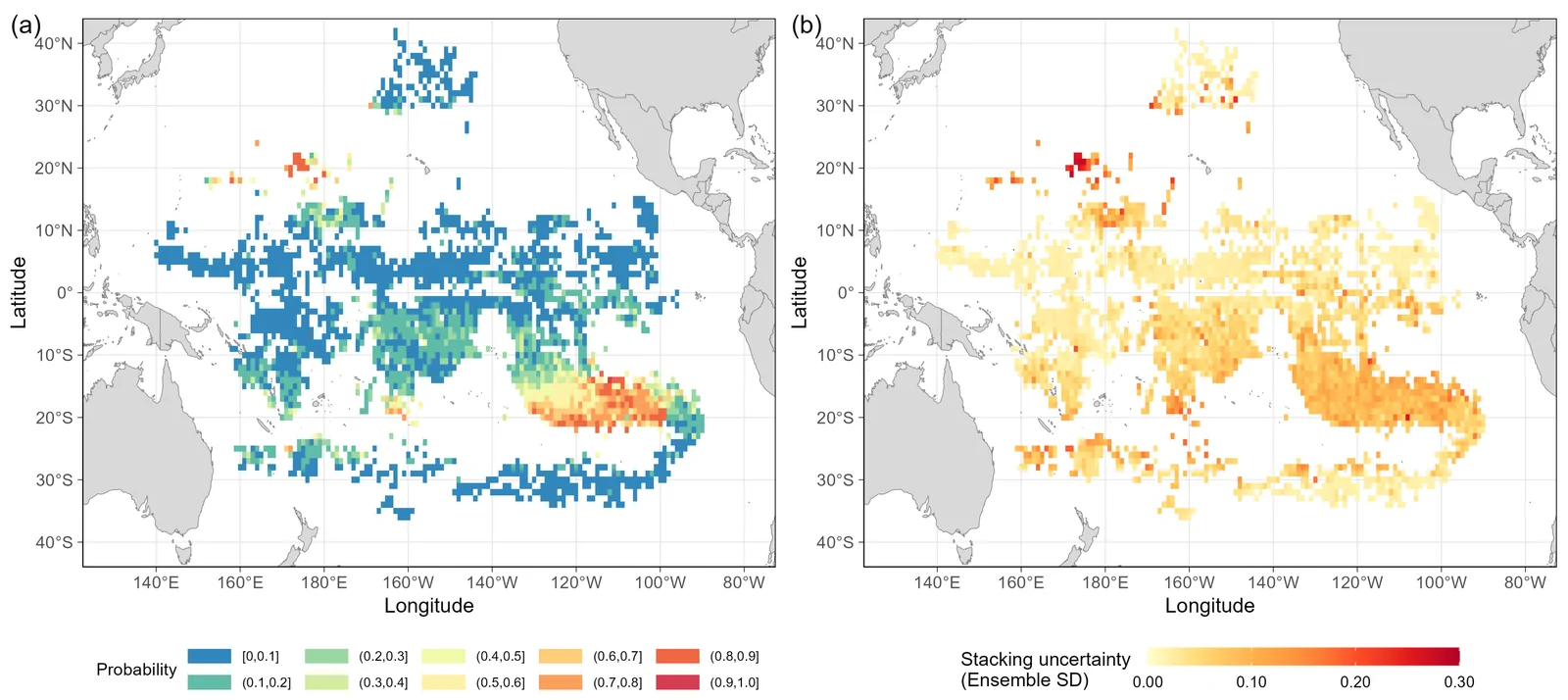
(**a**) Mean predicted habitat suitability and (**b**) base-model disagreement for *Tetrapturus angustirostris* within 1° × 1° grid cells containing observed longline sets, based on the stacking ensemble model. Predictions were generated only for observed fishing locations and averaged within grid cells.

Predicted habitat-suitability patterns were broadly similar between RF and the stacking ensemble. High-suitability habitat for shortbill spearfish was spatially dispersed across the Pacific but locally concentrated in several regions. The principal high-suitability hotspot was located between 100° W and 130° W and between 10° S and 25° S. Potentially suitable habitat was also predicted near 160° E–180° E and around 20° N (Figure 7a or Figure 8a). The RF model showed higher prediction ambiguity mainly around the periphery of suitable habitat, particularly near 115° W, 26° S and around 165° E–180° E (Figure 7b). In the stacking ensemble, base-model disagreement was also concentrated mainly in marginal or transition areas, with the highest values near 170° E–175° E and approximately 20° N (Figure 8b).

**Figure 9:**
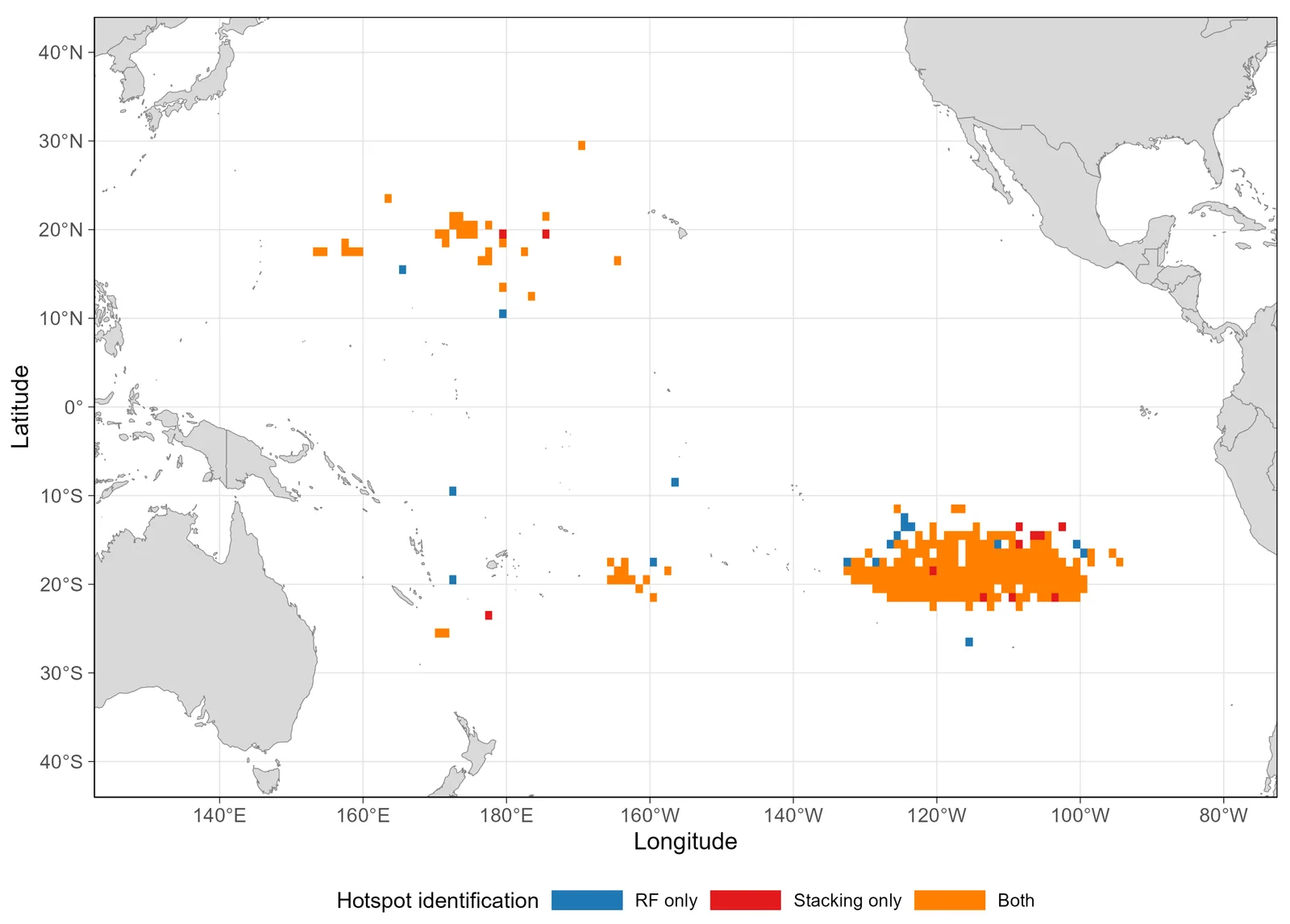
Spatial overlap of high-suitability habitat for *Tetrapturus angustirostris* predicted by the RF and stacking ensemble models.

**Figure 10:**
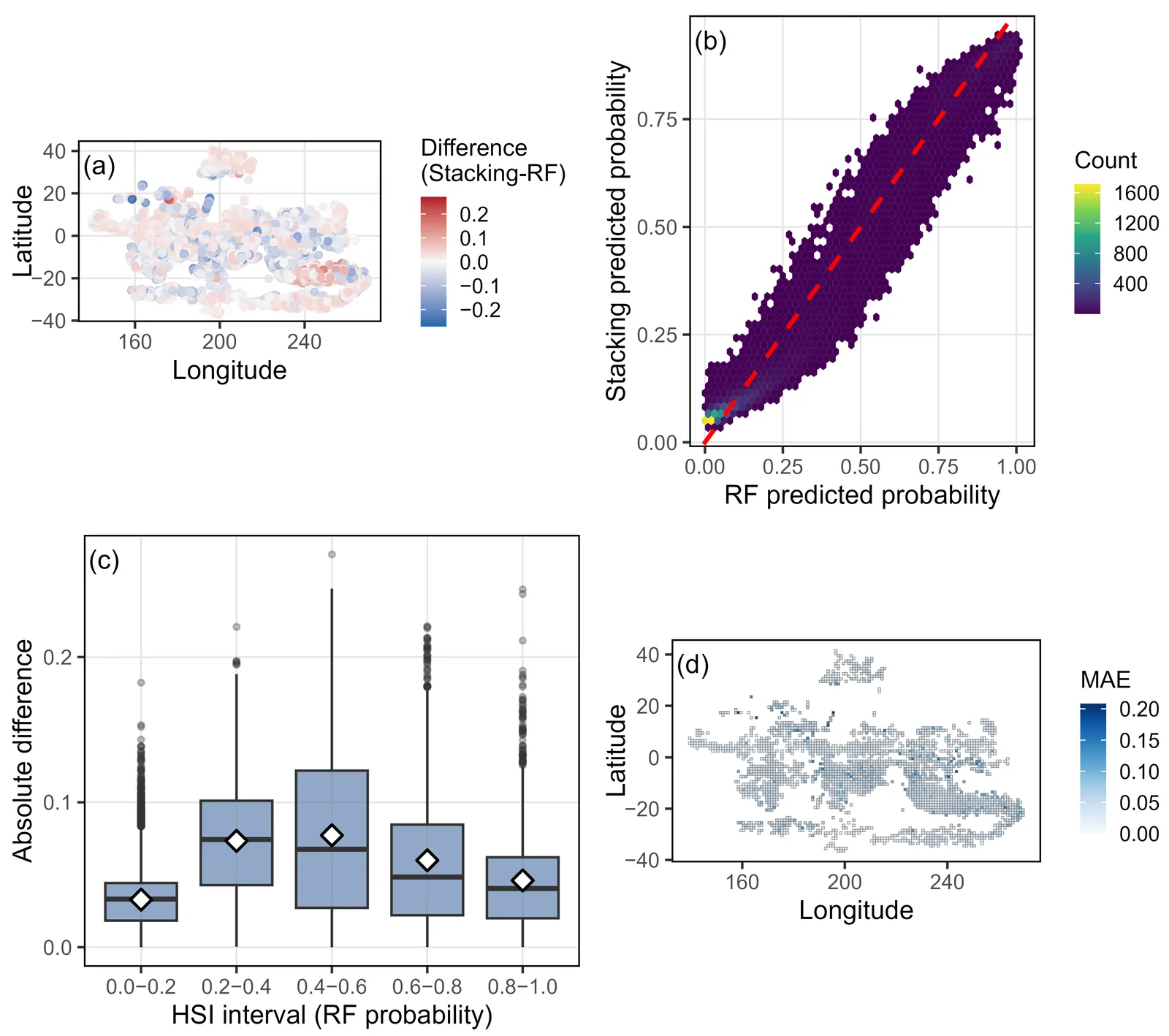
Integrated comparison of habitat-suitability index (HSI) from the Random Forest (RF) and stacking ensemble models. (**a**) Spatial distribution of prediction differences (Stacking—RF). (**b**) Correlation between RF and Stacking predicted probabilities; the red dashed line represents the 1:1 line of perfect agreement. (**c**) Boxplot of absolute differences across HSI intervals. Boxplots show the median (horizontal line), interquartile range (IQR; box), and 1.5 × IQR (vertical whiskers). Outliers beyond the whiskers are shown as open circles. White squares indicate mean values. (**d**) Spatial distribution of mean absolute error (MAE) at 1° grid resolution.

Predictions generated by the RF and stacking models were highly similar (Figure 9 and Figure 10a), as indicated by Schoener’s D (0.980) and Pearson’s correlation coefficient (r = 0.983) (Figure 10b). Pixel-level suitability estimates were closely correlated between the two models, whereas prediction differences were mainly concentrated within the moderate-suitability range (0.4–0.6). Differences were limited in high-suitability areas (≥0.6) (Figure 10c). High MAEs were primarily located near the boundaries of the study area and in regions characterized by steep environmental gradients, whereas prediction differences within the core distribution area were comparatively small (Figure 10d).

The high-suitability areas predicted by RF and stacking were similar in extent (RF: 37.866 million km^2^; stacking: 38.321 million km^2^) and showed an overlap of 92.4% (Figure 9). The centroids of the predicted high-suitability areas were separated by 178.7 km, with the stacking-derived centroid located slightly southwest of that predicted by RF.

### 3.6. Effects of Observer Sampling Effort on Model Predictions

Including log-transformed observed hooks resulted in only minor improvements in predictive performance. For RF, AUC increased from 0.869 to 0.873 and TSS from 0.584 to 0.589, whereas for stacking, AUC increased from 0.872 to 0.877 and TSS from 0.591 to 0.597. Predictions from models with and without observed hooks remained highly correlated (RF: Pearson r = 0.977, Spearman ρ = 0.961; stacking: r = 0.978, ρ = 0.967). Spatial similarity was also high, with Schoener’s D values of 0.925 for RF and 0.938 for stacking. The Jaccard overlap of high-suitability habitat was 0.931 and 0.940, respectively. Overall, incorporating observed hooks slightly improved model performance but did not materially alter the main habitat-suitability patterns.

## 4. Discussion

Model choice influenced predictive performance, the inferred importance of environmental variables, and the shapes of environmental response curves for shortbill spearfish habitat suitability. Overall, the stacking ensemble achieved the highest predictive performance, whereas RF outperformed the AUC- and TSS-weighted ensembles on some metrics and was the best-performing individual model. Despite differences in inferred variable importance and response relationships, both approaches identified broadly similar high-suitable habitat patterns. High-suitability habitat was concentrated in deep offshore waters of the subtropical southeastern Pacific (10° S–25° S, 100° W–130° W). Both models also predicted elevated suitability in the subtropical western North Pacific (160° E–180° E, 15° N–25° N), although observer coverage in this region was relatively sparse.

### 4.1. Ecological Interpretation of Environmental Predictors

SSS was identified as the most influential predictor in both the RF and stacking models, indicating a strong statistical association between predicted habitat suitability and relatively high-salinity offshore environments. Although SSS can influence osmotic regulation in fishes [41], highly migratory billfishes are unlikely to respond to SSS as an isolated factor at the basin scale. A more plausible interpretation is that SSS functions as an indicator for water-mass structure, stratification, and the relative stability of subtropical oceanic habitats [42,43]. The high-suitability range around 35–36 psu is consistent with subtropical water masses, which are generally clearer, more oligotrophic, and more vertically stratified than equatorial or coastal waters. Therefore, the importance of SSS is more appropriately interpreted as reflecting an association between shortbill spearfish occurrence and stable offshore water masses, rather than a direct physiological preference for high SSS.

This interpretation also helps explain why our results partly differ from studies that identified SST, mixed-layer depth, or DO as the primary drivers of billfish distribution [42,44,45]. Many previous studies focused on vertical habitat use, larval habitat, or regional-scale distribution, where temperature and oxygen directly constrain swimming performance and depth occupancy [46,47]. In contrast, SSS in the present study may better summarize the broad hydrographic setting of the fishing grounds. Compared with striped marlin in the eastern Pacific [48], the stronger association of shortbill spearfish with higher-SSS offshore waters suggests that closely related billfish species may differ in their use of water masses and productivity regimes, although this hypothesis requires confirmation using tagging or physiological data.

Spatially, the predicted high-suitability areas were concentrated in pelagic regions influenced by subtropical high-SSS water masses, further suggesting that water-mass structure may be associated with the large-scale distribution of shortbill spearfish. By contrast, lower-SSS equatorial waters may be associated with reduced suitability through differences in surface-water properties and stratification [43,49]. These patterns should be interpreted as statistical associations between water-mass environments and species occurrence within the sampled fishing domain, rather than evidence that SSS directly determines habitat suitability. The underlying ecological mechanisms, as well as their implications for population connectivity, require validation using tagging, genetic, or behavioural data.

DO showed a unimodal response, with suitability peaking at moderate concentrations rather than increasing monotonically. This pattern is ecologically meaningful because oxygen availability interacts with temperature to determine aerobic scope, swimming capacity, and accessible vertical habitat in large pelagic predators [50,51]. Low-oxygen waters can compress vertical habitat by restricting access to deeper layers, increasing overlap between pelagic predators and surface or near-surface longline gear [52,53]. However, very high surface DO may also correspond to colder or different hydrographic regimes that are not necessarily optimal for shortbill spearfish. The observed DO response should therefore be interpreted as the outcome of coupled oxygen, temperature, and water-mass gradients. This mechanism is particularly relevant under climate-driven ocean warming and deoxygenation, which are expected to modify oxygen minimum zones and potentially shift suitable habitat for tropical pelagic fishes [52,53].

Chl was negatively associated with predicted suitability, indicating that shortbill spearfish occurrence in the observed fishery was more closely linked to oligotrophic offshore environments than to highly productive surface waters. Subtropical gyres are typically characterized by low Chl, low productivity, and strong stratification [54], whereas mesoscale processes can create localized foraging opportunities within an oligotrophic background [55]. Similarly, SSH may represent mesoscale dynamic structure rather than a direct habitat requirement. Overall, the combined effects of low Chl, intermediate SSH, offshore distance, and bathymetric depth suggest that shortbill spearfish habitat is shaped by a mixture of water-mass background, prey-field organization, and access to oceanic foraging structures.

### 4.2. Model Differences

The superior performance of the stacking ensemble can be explained statistically by its ability to combine complementary prediction functions from multiple algorithms. Individual models differ in how they represent nonlinearity, interactions, thresholds, and extrapolation. RF can capture complex interactions and local nonlinearities, whereas GLM and GAM provide smoother parametric or semi-parametric responses, and XGBoost can emphasize sequentially improved tree structures [56]. Stacking uses a meta-model to learn how these base-model predictions should be combined, thereby reducing dependence on any single algorithm and potentially lowering algorithm-specific bias [57]. This interpretation is consistent with ensemble-forecasting theory, which suggests that ensembles may improve generalization when component models capture partly different structures in the data [9,10,58].

Although stacking achieved the highest AUC and TSS, its advantage over RF was small in absolute terms. Thus, the statistical improvement should be viewed as incremental rather than as evidence that stacking reveals a fundamentally different ecological niche. The strongest ecological support comes from the convergence between RF and stacking in identifying similar high-suitability areas in the subtropical southeastern Pacific and a secondary area in the central North Pacific. By contrast, differences between models were concentrated mainly in moderate-suitability and marginal zones, where small changes in predicted probability can affect the delineation of transition areas but do not substantially alter the inferred core habitat pattern [59,60].

### 4.3. Limitations and Uncertainty

Although the models identified consistent habitat patterns and key environmental associations, these findings should be interpreted in light of several limitations. First, because the analysis was based on fishery observer data, predicted habitat suitability may partly reflect the spatial distribution and sampling structure of the observed longline sets in addition to underlying ecological habitat associations. Observers randomly selected a proportion of baskets from each set for catch observation and biological measurements, and the number of observed hooks therefore reflected observer sampling effort based on this sampling procedure. Although observed hooks were not included in the primary models, the sensitivity analysis showed that incorporating log-transformed observed hooks resulted in only minor improvements in RF and stacking performance and did not materially alter the main spatial predictions. Nevertheless, some influence of observer sampling effort on encounter probability may remain, and the predicted probabilities should therefore be interpreted within the observed fishery sampling framework rather than as effort-standardized ecological occurrence probabilities [60].

Second, although repeated random cross-validation was useful for comparing model performance, it may not fully account for spatial or temporal dependence among observations and could therefore overestimate predictive performance for spatially structured ecological data [57].

Third, the use of monthly environmental data may have overlooked finer-scale oceanographic variability that influences short-term habitat selection and fishery interactions [61,62]. In addition, the predicted habitats were not independently validated using tagging, fishery-independent surveys, or genetic data. Therefore, the results should be interpreted as species–environment associations within the sampled fishing domain rather than as definitive estimates of the species’ full ecological distribution [60].

### 4.4. Management Consideration

The predicted habitat maps provide a spatial basis for identifying potential bycatch-risk areas, but they should not be used alone to define management measures. Bycatch risk depends on both species habitat suitability and the spatial–temporal distribution of fishing effort. Therefore, the most direct management application is to combine the habitat-suitability surface with longline effort data to identify areas where high suitability overlaps with intensive fishing. Such risk maps could inform targeted observer deployment, electronic monitoring priorities, spatially explicit bycatch assessment, and the design of precautionary mitigation measures. Areas consistently identified as highly suitable by both RF and stacking may be treated as high-confidence candidate risk areas, whereas model-divergent marginal areas may be appropriate for adaptive monitoring.

The results also have relevance for marine spatial planning and dynamic ocean management. Because the environmental features associated with shortbill spearfish habitat are dynamic, static spatial measures alone may be insufficient to manage bycatch risk efficiently. Dynamic management approaches that update risk information using near-real-time oceanographic data have been proposed to reduce bycatch while maintaining fishing opportunities [63,64]. The present habitat models could contribute to such approaches if combined with operational fishing-effort data and near-real-time environmental products. In this context, habitat maps should be considered decision-support layers rather than fixed conservation boundaries.

Climate-driven oceanographic changes may alter the suitability and location of shortbill spearfish habitat. Ocean warming, deoxygenation, changes in stratification, and shifts in subtropical gyre structure could modify the salinity, oxygen, and productivity regimes associated with high suitability [52,53,65]. For highly migratory pelagic fishes, such changes may shift species distributions and redistribute interactions with fisheries [65,66]. Future work should therefore develop seasonal and interannual prediction frameworks, evaluate habitat suitability under climate scenarios, and test model transferability to other ocean basins, especially the Indian Ocean. Integrating habitat models with biotelemetry, population genetics, and climate projections would help determine whether the environmental associations identified here represent region-specific fishery encounters or broader ecological preferences of shortbill spearfish.

## 5. Conclusions

This study provides a comparative modelling framework for characterizing the habitat suitability of shortbill spearfish in the Pacific Ocean using fishery observer data. By comparing individual algorithms with ensemble strategies, we showed that model choice can influence both predictive performance and the interpretation of species–environment relationships. Under the present dataset and modelling framework, the stacking ensemble achieved the highest overall predictive performance, while RF provided useful information on dominant environmental predictors. Therefore, the results do not imply that stacking is universally superior for habitat modelling, but rather indicate that ensemble approaches may improve predictive stability when applied to sparse and fishery-dependent occurrence data.

The predicted habitat patterns suggest that shortbill spearfish occurrence in the Pacific is strongly associated with subtropical oceanic environments characterized by high SSS, low Chl concentration, moderate DO levels, and dynamic offshore conditions. These findings extend current understanding of the environmental associations of a poorly studied billfish species and provide spatially explicit information that can support bycatch-risk assessment in pelagic longline fisheries. However, these maps should be interpreted as probabilistic habitat-suitability estimates within the sampled fishing domain, rather than as the realized or complete distribution of shortbill spearfish.

Future research could further improve this framework in several directions. First, independent validation using additional observer datasets, fishery-independent surveys, or electronic monitoring records is needed to evaluate the robustness of the predicted suitable habitats. Second, temporal prediction should be strengthened by incorporating seasonal and interannual environmental variability to assess whether habitat suitability shifts under changing oceanographic conditions. Third, model transferability should be tested in other ocean basins, particularly the Indian Ocean, to determine whether the environmental associations identified in the Pacific are region-specific or represent broader ecological preferences of shortbill spearfish. Integrating biotelemetry, population genetics, and finer-resolution environmental data would further help clarify the mechanisms underlying core habitat formation, movement connectivity, and bycatch exposure.

## Figures and Tables

**Table 1:** Environmental predictor variables used in habitat-suitability models.

Variables (Abbreviation)	Units	Spatial Resolution	Temporal Resolution
Sea surface temperature (SST)	°C	0.25°	Monthly
Sea surface salinity (SSS)	psu	0.25°	Monthly
Sea surface height (SSH)	m	0.25°	Monthly
Mixed layer thickness (MLT)	m	0.25°	Monthly
Chlorophyll-a (Chl)	mg/m^3^	0.25°	Monthly
Oxygen concentration (DO)	mmol/m^3^	0.25°	Monthly
Bathymetric depth (Depth)	m	5 arc-minute	-
Distance to the nearest land (Land_dis)	10^3^ km	5 arc-minute	-

**Table 2:** Variance inflation factors for explanatory variables.

Explanatory Variable	Original VIF Value	Adjusted VIF Value
SST	23.76	-
SSS	1.32	1.27
SSH	3.25	3.09
MLT	1.32	1.30
Chl	1.60	1.31
DO	20.10	2.67
Depth	1.22	1.20
Land_dis	2.01	2.00

## Data Availability

Data may be made available by the corresponding author upon reasonable request and with permission from the data provider.
